# Molecular Epidemiology of HCV RNA Genotype-3 in Dhaka City, Bangladesh

**DOI:** 10.1055/s-0043-1771182

**Published:** 2023-08-07

**Authors:** Md Arifur Rahman, Md Monirul Islam, Md Eunus Ali, Mohammad Ariful Islam, Farhana Afroze, Mohammad Shamim Hossain, Ahmed Abu Rus'd

**Affiliations:** 1 Department of Microbiology, Jagannath University, Dhaka, Bangladesh; 2 PCRLaboratory, Ibn Sina Diagnostics and Imaging Center, Dhaka, Bangladesh; 3 PCR & Molecular Diagnosis Section, Labaid Limited (Diagnostics), Dhaka, Bangladesh; 4 Department of Microbiology and Immunology, BSMMU, Dhaka, Bangladesh; 5 Department of Biology, University of Copenhagen, Copenhagen, Denmark; 6 Division of Molecular Diagnosis and Clinical Genetics, BioIcon Academy, Dhaka, Bangladesh

**Keywords:** genotypes, cirrhosis, hepatocellular carcinoma, reverse transcription, cDNA, antiviral therapies, viral load

## Abstract

Hepatitis C virus (HCV) is a causative agent that causes chronic liver diseases worldwide. It is a little, enclosed, single-stranded ribonucleic acid (RNA) virus. The recognition of the pathogenic HCV genotype is critical for the remedy of its sufferers. The aim of this study was to identify the HCV RNA genotype to decide the correct treatment of hepatitis C positive sufferers in Bangladesh. Blood samples were collected from 390 individuals and isolated RNA (60 µg) from blood plasma. Extracted RNA was used for quantitative HCV RNA, and complementary DNA (cDNA) was prepared by polymerase chain reaction (PCR) conducted by reverse transcriptase enzyme. This cDNA amplified in multiplex by RT-PCR, which was performed with specific set of primers. The HCV RNA genotype was detected 297 of 390 patients. Of the 390 test samples, 200 (51.28%) samples were from males and 190 (48.71%) were from females, with age ranging from 5 to 78 years. In all, 166 of 200 male samples and 131/190 female samples were found positive for HCV. Of these 390 participants included in the study, 213 (54.61%) were identified as genotype 3 positive, 78 (20%) as genotype 1 positive, 6 (1.53%) as genotype 6 positive, and the remaining 93 (23.85%) samples were unclassified due to low/undetected viral load. In this study, we detected the highest percentage (30.89%) of genotype 3 HCV in patients aged 51 to 60 years. The results suggested that genotype 3 HCV is frequently present in Bangladesh and it is usually responses better to interferon therapy. However, genotype 1 and 6 HCV have also been found circulating in this country, which demands longer treatments and effective control measures.

## Introduction

We know hepatitis C virus (HCV) is a causative, acute, and chronic inflammation in the liver by viral infection. According to World Health Organization (WHO), approximately 1.4 million patients die yearly by hepatitis infection, cancer, and liver cirrhosis. 1 2 Every year, approximately 170 million persons are severely infected and 3 to 4 million humans are infected newly by HCV. 3 The superiority of HCV contamination varies worldwide by locality and ethnicity. 3 The taint of HCV is notably higher in some nations and regions, which include Europe with approximately 25 million HCV positive cases. 4 In Bangladesh, the actual pattern of the existing genotype has not been properly studied, but it was mentioned to be 0.6% within the rural population 5 and in the published literature the figure is approximately 0.2 to 1% within the general population. 6 In another observation, it was found that HCV infection could be very excessive among drug addicted people (PWID), 7 8 but in 2011 it was recorded that HCV infection rates have grown to 95.7% in a northwestern part and 39.6% in Dhaka city. 9 Consequently, HCV stays an essential cause of morbidity and mortality in Bangladesh. 6 Even though several risk factors HCV infections have been found in 50% of cases, no major reasons for transmission can be considered in the remaining 50% of cases. 10 However, the main causes for transmission of HCV in Bangladesh is from quacks, shaving, dialysis procedure, haircut in barbershops, body piercing, dental procedures, intravenous injection, etc. 11 HCV shows an excessive genetic diversity both for HCV vaccine development and for genotypic drug development. 12 13 The updated geographic HCV genotype flow in the world is very complex. Curiously, it seems that genotypes 1, 2, and 3 (especially 1a, 2b, 2a, and 3a) are distributed widely around the world and other genotypes are confined in some geographic locations. 14 15 16 It is far obtrusive that both HCV and HBV are major global health issues and they are swiftly spreading in developing countries due to lack of health education, poverty, illiteracy, vaccination, etc. 17 Genotype records of HCV infection is important to produce personalized treatment and mitigate successful patient outcome for useful treatment type, length, and doses of medicine. 18 19 Genotype study for HCV infection in a large population of a country is quite difficult. In Bangladesh, some studies have been published to show HCV genotype, prevalence, risk factors, etc., but developing exact treatment strategies from these studies has not been practical. This study aims to identify the functional HCV ribonucleic acid (RNA) infection. In addition, we can know which HCV genotype is spreading within the selected place. It will also help the doctor to prescribe more precise and suitable treatment for HCV infection and implement pointers to provide customized medicine.

## Materials and Method

### Study Population

In this study, we were selected 390 HCV positive individuals. These patients were identified by an anti-HCV screening test. The study period was from January 2019 to December 2021. The blood samples were collected from distinctive branches of Ibn Sina Diagnostics and Imaging center, Dhanmondi, Dhaka, Bangladesh. Blood samples of 5 mL were taken in a Lavender-top vacutainer tube (EDTA) from the patients. Plasma was separated from blood within 6 hours of collection and was stored at –20°C until further testing. While collecting blood samples, the patient's history was also collected for clinical information database.

### Extraction of RNA and cDNA Synthesis

Hepatitis C is a ribonucleic acid (RNA) virus. First, we extracted viral RNA from blood plasma. The extraction kit was QIAsymphony DSP Virus/pathogen kit. The manufacturer's extraction guidelines were followed wherever the input volume of plasma/serum was 200 µL, and 60 µL of purified RNA was eluted.

### HCV Genotype with Specific RT-PCR

HCV genotyping was done with genotype-specific primers. For the targeted sequence of the HCV genome, it was done for subtype 1a and six genotypes of HCV (1, 1a, 2, 3, 4, 5a, and 6). 20 For each sample, three separate reaction mixtures were made (polymerase chain reaction [PCR] tubes1, 2, and 3), whereas PCR tube 1 was for HCV detection and also HCV genotypes 5a and genotype 1 detection, PCR tube 2 for HCV genotypes 1a, 4, and internal control (IC), and PCR tube 3 for HCV genotypes 3, 2, and 6 detections. PCR tubes 1, 2, and 3 were added with 10 µL multiplex master mix and 10 µL of HCV primer-probe mix 1, 2, and 3 were added. Then, 5.0 µL of cDNA were added in each tube and was adjusted to the final volume of 25.0 µL in each PCR tube. Real-time PCR system Rotor-Gene Q MDX 5plex HRM was used and reporter dye green was selected for tube 1, orange for tube 2, and yellow for tube 3. For PCR reaction operation, initial denaturation step was done at 94°C for 10 seconds, then the PCR protocol defined the thermal profile as follows: 40 cycles; 94°C for 15 seconds for denaturing; 58°C for 45 seconds for annealing; and 72°C for 15 seconds for extension.

### Data Interpretation

HCV genotyping is a qualitative assay. In case of two genotype amplifications, earlier cycle threshold (CT) value genotype was considered as the dominant genotype and positive result (CT value <37; Table 1 ).

**Table 1:** Interpretation of genotype between reporter dye and PCR tube: one sample loaded into separate three tubes and reporter dye. If one or multiple reporter dyes showed a signal curve, the sample indicated the following genotype-specific HCV

Tube no	Reporter dye	Signal	HCV genotype
1	Green	Present	HCV
	Yellow	Present	5a
	Orange	Present	1
2	Green	Present	1a
	Yellow	Present	4
	Orange	Present	IC
3	Green	Present	3
	Yellow	Present	2
	Orange	Present	6

Abbreviation: HCV, hepatitis C virus; IC, internal control; PCR, polymerase chain reaction.

## Results

**Fig. 1:**
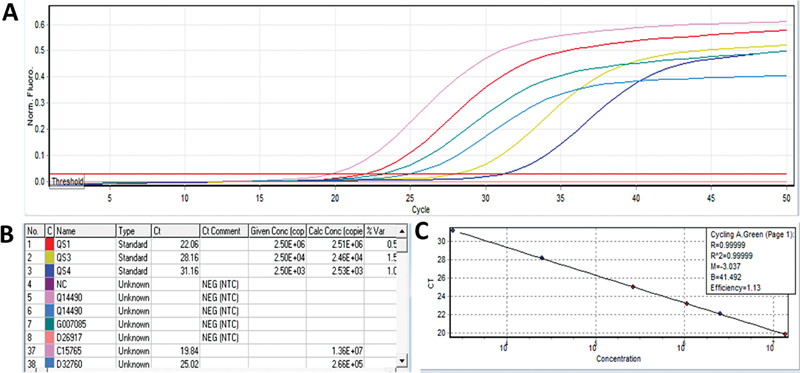
( **A–C** ) Hepatitis C virus (HCV) viral load analysis in Rotor-Gene Q Multiplex polymerase chain reaction (PCR).

Fig. 1A shows the reporter dye signal curve, four curves for four standards and another two curves for unknown samples. Fig. 1B shows the comparison between the given and calculated concentrations; Fig. 1C shows the standard curve for calibrators and samples.

**Fig. 2:**
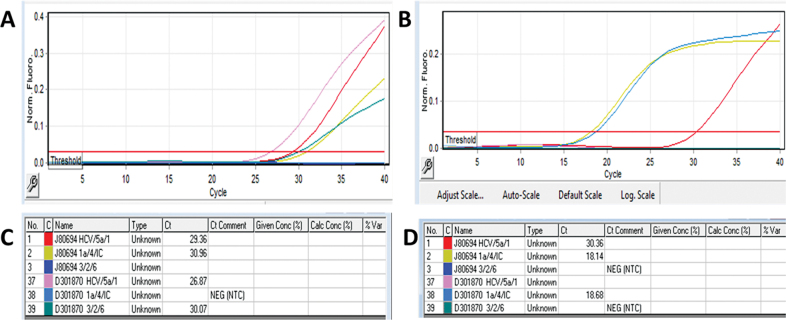
( **A–D** ) Hepatitis C virus (HCV) genotyping analysis in Rotor gene Q HRM polymerase chain reaction (PCR).

Fig. 2A, C shows HCV positive ( *red color* ) and genotype 3 ( *green color* ); Fig. 2B and D shows HCV positive ( *red color* ) and genotype 1 ( *light blue color* ).

### Prevalence of HCV Genotyping in Studied Population

Table 2 represents the percentage of genotype in our studied samples. Of the 390 patients, males comprised 57% of the patients and females comprised approximately 42%. In 390 tested samples, classified PCR fragments were seen in 297 (76.15%) samples, whereas unclassified genotypes were found in 93 (23.85%) samples.

**Table 2:** Observation of HCV genotype in between infected male female patients ( *n* = 390)

Genotype	Subtype	Male (%)	Female (%)	Total (%)
	1a	42 (18.83%)	21 (12.57%)	63 (16.15%)
	1b	9 (4.04%)	6 (3.59%)	15 (3.85%)
3		111 (49.78%)	102 (61.08%)	213 (54.61)
6		4 (1.79%)	2 (1.98%)	6 (1.53%)
Unclassified		57 (25.56%)	36 (21.56%)	93 (23.85%)
Total		223	167	390

Abbreviation: HCV, hepatitis C virus.

The diversity of classified genotyping is mentioned as the following: genotype 1a accounted for 63 (16.15%) cases, type 1b for 15 (3.85%), type 3 for 213 (54.61%), and genotype 6 for 6 (1.53%) cases. The predominant genotype of this study was type “3” (54.61%), followed by type “1” (20%), ‘1a” (16.15%), “1b” (3.85%), and “6” (1.53%). The predominant genotype 3 was found in 111 (49.78%) males, followed by genotype 1 (22.87%), genotype 6 (1.79%), and undetermined (25.56%). Correspondingly, the most common genotype found in the infected female samples was type 3 (61.08%), followed by genotype 1 (16.16%), genotype 6 (1.98%), and undetermined (21.56%). In males, the HCV subgenotype pattern “1a” was detected in 42 (18.83%) males and “1b” was detected in 9 (4.04%) HCV infected males. The following were detected in female patients: subtype “1a” in 21 (12.57%) and “1b” in 6 (3.59%) patients. Genotype 3 was found to infect males and females approximately equally, whereas genotype 1a was found to be higher in males.

### Classification of HCV Genotypes in Various Age Groups

The classification of HCV genotype in 390 patients according to various age groups is described in Table 3 . In this study, type 3 was found to be the highest in people aged 51 to 60 years, which was 30.98% of the total patients. The second highest infection for type 3 was found in the age 41 to 50 years age group. Genotype 1a was the predominant type in older patients (age >60 years), accounted for 42.86% of cases. Type 6 was found in the lowest percentage of the patient in all age groups.

**Table 3:** Percentage of HCV genotypes in various age groups (samples = 390)

Genotypes	21–30 y	31–40 y	41–50 y	51–60 y	> 60 y
** 1a**	3 (4.76%)	3 (4.76%)	24 (38.09%)	6 (9.52%)	27 (42.86%)
** 1b**	0	3 (20%)	7 (46.67%)	2 (13.33%)	3 (20%)
** 3**	15 (7.04%)	21 (9.86%)	60 (28.17%)	66 (30.98%)	51 (23.94%)
** 6**	0	3 (50%)	0	1 (16.67%)	2 (33.33%)
** Unclassified**	24 (25.81%)	9 (9.68%)	12 (12.90%)	39 (41.94%)	9 (9.685%)
** Total**	42 (10.77%)	39 (10%)	103 (26.41%)	114 (29.23%)	92 (23.59%)

Abbreviation: HCV, hepatitis C virus.

### HCV RNA Viral Loads Found in Male and Female Samples with Various Genotypes

The copies of HCV for type-specific genotype in the samples of male and female patients are shown in Table 4 . There were classes categorized according to the level: low (<600,000 IU/mL), intermediate (60,0000–800,000 IU/mL), and high (>800,000 IU/mL) viral copies (1 IU = 4 copies of HCV RNA).

**Table 4:** HCV viral load was classified in gender and genotype of the studied people

Genotype	Viral load (IU/mL)			*p* value
	<600,000	600,000–800,000	>800,000	
Genotype 3	104 (48%)	15 (7.04%)	94 (44.13%)	<0.07 a
Other genotypes	57 (67.85%)	7 (8.33%)	20 (23.80%0	
Male	78 (48.45%)	14 (63.63%)	79 (69.29%)	<0.03 a
Female	83 (51.55%)	8 (36.36%)	35 (30.70%)	

Abbreviation: HCV, hepatitis C virus.

a *p* value calculation was done by Student's *t* -test.

Viral load was found significantly high ( *p* < 0.07) in pretreatment samples infected with HCV genotype 3 when compared with other genotypes. It was observed in male and female samples that viral load was high and significant ( *p* < 0.03) in HCV-infected patients.

### Minimum Viral Load among Tested Samples

**Fig. 3:**
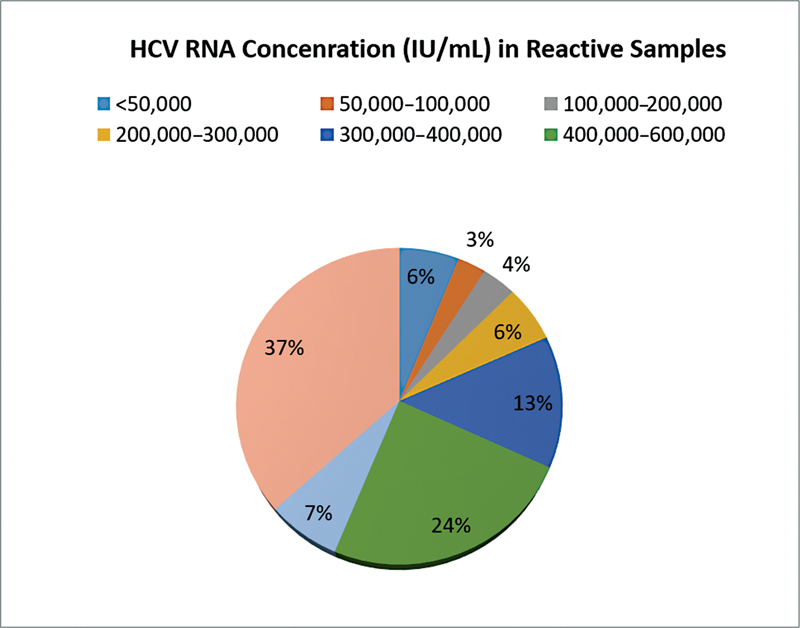
Minimum viral load count among the samples. HCV, hepatitis C virus; RNA, ribonucleic acid.

HCV RNA genotype was detected in 297 individuals and viral load test measure in IU/mL, so sensitivity was estimated based on this. The minimum viral loads were observed for 19 samples at <50,000 IU/mL, whereas the majority of the samples ( *n* = 114) were found to be at >800,000 IU/mL ( Fig. 3 ).

## Discussion

The clinical consequences of different HCV infection patients have been found to fluctuate from acute hepatitis disease to liver cirrhosis. 21 Documentation of the HCV genotype is essential for research of numerous features of HCV infection, which include pathogenesis, epidemiology, and various therapy response. 22 23 An appropriate and reliable HCV genotyping methods depend on epidemiological and experimental test in a large scale. 24 A number of laboratory protocols have been described for the detection of HCV genotypes. For this case, PCR was used broadly, which requires a type-specific primer (amplification of different genotypes) for amplification of viral RNA of clinical samples. 25 26 RT-PCR is extensively used for the diagnosis of HCV in comparison to other existing bioassays. 27 Our data showed that genotype 3 (54.61%) and genotype 1a (16.15%) were detected in the studied patients. Genotype 3 was found in the highest percentages in patients aged 51 to 60 years among the tested patients, accounting for 30.98% of total patients. According to some studies, regarding the occurrence of HCV genotype, genotype 3 is more prevalent in Asian countries like Pakistan and Iran. 28 Type 3 HCV variants and six subtypes were found in Asia, whereas subtypes 1, 2, and 4 were found in Africa. It suggests that existing types and their similar and diverse subtypes found in different regions have been circulating for a long time in the local community. HCV prevalence is variable in different areas of the universe and diversified in various groups of the locality. For example, HCV genotype “1a” is mostly found in the United States and northern part of Europe, while HCV genotype 1 is mostly found throughout the world, accounting for about 83.4 million cases (42.6% of the total HCV cases), of which one-third are found in East Asia. 29 Furthermore, several studies confirmed that genotypes 1a and 1b are highly prevalent in HCV infected patients around various part of the world. 30 31 Prevalence of type 3 findings are consistent with the findings in previous studies that genotype 3 is one of the most collective genotypes in Bangladesh. 7 We found 15 (7.04%) patients with genotype 3 with an “intermediate viral load” (600,000-800,000 IU/mL) after HCV RNA quantification.

Research revealed that patients having a high viral load (>800,000 IU/mL) with genotype 3 needs to be treated for 24 weeks, whereas patients with low RNA viral load (<600,000 IU/mL) needs to be treated for 16 weeks. These patients’ HCV RNA PCR reports are undetermined at week 4 of treatment. 30 Therefore, the HCV genotype and basal RNA viral load are important for scheduling the therapy against HCV. The result of this study showed that type 3 HCV genotypes are common in Bangladesh and could impressively respond to interferon therapy, whereas types 1 and 4 require longer treatment. Appropriate preventive measures should be additionally considered to control spreading of this disease.

## Conclusions

Our study emphasized the prevalence of HCV genotype in Bangladesh and that HCV genotype 3 is the more persistent and predominant type. As genotype has a direct relation with the duration of the treatment in severe cases of hepatitis C infection, this study will be helpful in choosing the best treatment approach for this dreadful disease. The national difference is also present in HCV genotype. Here, the maximum number of infection is found people between 51 and 60 years, with a male predilection. Viral load (baseline) is significantly elevated in patients affected by HCV genotype 3 compared to those affected by genotypes “1” (subtype a and b) and “6” and unclassified genotype. The highest number of samples ( *n* = 114) were detected at >800,000 IU/mL and the minimum number of samples ( *n* = 19) were detected at <50,000 copies/mL. Therefore, to figure out the exact genotype of the national population besides widespread clinical data collection, a national survey with HCV genotyping may be the prime source for determining the etiology and/or present consequences of HCV genotype circulation here.
